# SARS-CoV Antibody Prevalence in All Hong Kong Patient Contacts

**DOI:** 10.3201/eid1009.040155

**Published:** 2004-09

**Authors:** Gabriel M. Leung, Pui-Hong Chung, Thomas Tsang, Wilina Lim, Steve K.K. Chan, Patsy Chau, Christl A. Donnelly, Azra C. Ghani, Christophe Fraser, Steven Riley, Neil M. Ferguson, Roy M. Anderson, Yuk-lung Law, Tina Mok, Tonny Ng, Alex Fu, Pak-Yin Leung, J.S. Malik Peiris, Tai-Hing Lam, Anthony J. Hedley

**Affiliations:** * University of Hong Kong, Hong Kong Special Administrative Region (SAR), China;; † Department of Health, Hong Kong SAR, China;; ‡ Imperial College, London, England

**Keywords:** Seroprevalence, SARS, Hong Kong, SARS-CoV, dispatch

## Abstract

A total of 1,068 asymptomatic close contacts of patients with severe acute respiratory (SARS) from the 2003 epidemic in Hong Kong were serologically tested, and 2 (0.19%) were positive for SARS coronavirus immunoglobulin G antibody. SARS rarely manifests as a subclinical infection, and at present, wild animal species are the only important natural reservoirs of the virus.

Since severe acute respiratory syndrome (SARS) and the corona virus (SARS-CoV) that causes it have emerged and spread, considerable progress has been made in understanding the biology, pathogenesis, and epidemiologic features of both the virus and the disease. Epidemiologic studies of hospitalized patients suggest that the overall transmissibility of SARS (as indicated by the basic reproductive number *R_0_* = 2.7, 95% confidence interval [CI] 2.2–3.7) (*1*) is relatively low compared to other pathogens. However, such studies could not take into account possible episodes of mild or moderate illness that did not require inpatient medical care and could not address whether asymptomatic community spread played a role in the 2003 epidemic. If this type of spread occurred, sufficient herd immunity against SARS-CoV to protect against another large-scale outbreak might have been developed in the population. The full spectrum of disease associated with SARS-CoV infection should be examined to define more precisely what constitutes a case requiring quarantine and isolation to minimize potential human-to-human spread. Understanding these issues requires the systematic study of the seroprevalence of SARS-CoV antibody in a large sample stratified by age and other baseline characteristics, especially since children were disproportionately less affected by SARS, both in terms of reduced incidence and severity of infection. Serologic surveys can be based on a random sample from the total population with appropriate stratification, on serum collected for other reasons (e.g., blood donors, all hospital admissions), or on surveys of persons who resided in sites of superspreading events or who have had close contact with a confirmed SARS patient.

We report a serologic survey for immunoglobulin (Ig) G against SARS-CoV in a representative sample of close contacts of all SARS patients in Hong Kong (>76% had laboratory confirmation of SARS by either paired serology or repeat reverse transcription–polymerase chain reaction (RT-PCR) according to World Health Organization [WHO] criteria [2]).

## The Study

During the epidemic, close contacts were prospectively identified by the Hong Kong Special Administrative Region Government Department of Health through standardized telephone interviews with all 1,755 confirmed SARS patients within 1 week of hospital admission (February 15–June 22, 2003). A close contact was defined as a person who had cared for, lived with (in the same household), or came into direct contact with body fluids of the SARS patients within 10 days before hospital admission. A total of 3,612 close contacts were recorded; 505 were diagnosed as having SARS. Of the remaining 3,107 contacts, 2,805 (90%) had a telephone number available, as reported by the primary patient. We successfully contacted 2,337 (83%) from October 23 to November 30, 2003, and 1,776 (57% of those eligible) consented to a telephone interview after the purpose of the study was explained to them by trained public health nurses. The interview consisted of questions that assessed the relationship between the patients and contacts; the timing, intensity and frequency of contact; precautionary measures adopted during contact with the patient; known contact with other SARS patients; clinical symptoms of febrile, respiratory, gastrointestinal, or constitutional illness since February 2003; medical and travel history; and sociodemographic details. Participants were then invited to provide blood samples for serologic testing. Shopping coupons (worth U.S. $25.00) were given to participants after blood was collected as compensation for time and travel costs.

Samples were screened by the Government Virus Unit of the Department of Health by using viral lysate enzyme-linked immunosorbent assay (ELISA) (GBI Biotech, Beijing). Positive results were confirmed with immunofluorescence assay (IFA) and neutralization tests. For the IFA, microscopic slides coated with SARS-CoV–infected FRhK4 cells were incubated with serum samples at serial twofold dilutions starting from1:25. A positive test is indicated by cytoplasmic fluorescence under UV microscopy. By using IFA as the standard, the ELISA detects antibody with IFA titer of >25 (i.e., sensitivity of 100%) and has a specificity of 95%. Neutralization test was performed by standard virologic method with Vero E6 cells and SARS-CoV isolate 6109. A titer of >10 was considered positive. The reported sensitivity of 100% was for convalescent-phase serum samples taken a few weeks after the onset of infection in SARS patients, which should apply to our study. During the early phase of infection, IgM predominates; the ELISA kit we used detects IgG only. Therefore, the sensitivity is 80%–90% (depending on the number of days after illness onset when the serum samples were taken). However, this sensitivity should not have affected our findings, which were based on tests carried out at least 6 months after the last reported case of SARS in Hong Kong. The study received ethics approval from the Department of Health Ethics Committee, which complies with the Declaration of Helsinki.

### Results and Conclusions

Of the 1,068 samples analyzed, 2 (0.19%, exact 95% CI 0.02%–0.67%) had a positive titer (1:25 to 1:50 on IFA compared to at least 1:100 in most recovered SARS cases) for SARS-CoV IgG antibody. Neither participant with a positive sample reported a chronic medical condition or being sick with febrile or respiratory illness from February to August. Both seropositive participants arose from two superspreading events in Hong Kong, i.e., Prince of Wales Hospital nosocomial outbreak and Amoy Gardens environmental point source community outbreak (*1**,**3*). The contact of the Prince of Wales Hospital seropositive participant reported one other close contact, who was interviewed but declined to be tested. The other seropositive index patient living in Amoy Gardens was separately identified by three intrafamilial index patients, all of whom lived in the same household and reported only each other as close contacts. The participants who consented to testing were broadly similar to those who declined, except that the first group had relatively fewer children and fewer of the first group were men (Table). However, those who consented to testing were more likely to report more frequent contact and closer relationships with SARS patients, more febrile or respiratory illness episodes since February, and a travel history to SARS-affected regions, which may have biased our seroprevalence estimate upwards.

**Table:** Characteristics of close contacts recalled for serologic testing (N = 1,776)

Characteristic	Tested for IgG against SARS-CoV, n = 1,068 (%)	Declined antibody testing, n = 708 (%)	p value
Age (y)			< 0.001
<10	53 (5.0)	126 (18.1)	
11–17	77 (7.2)	68 (9.7)	
18–44	515 (48.3)	278 (39.8)	
45–64	330 (30.9)	138 (19.8)	
>65	92 (8.6)	88 (12.6)	
Sex			0.02
Female	579 (54.2)	341 (48.3)	
Male	489 (45.8)	365 (51.7)	
Travel history to SARS-affected areas since February, 2003^a^			< 0.001
Yes	523 (49.0)	268 (37.9)	
No	545 (51.0)	440 (62.1)	
Relationship with SARS case			0.001
Household family member	789 (74.4)	499 (70.5)	
Non-household family member or relative	230 (21.7)	164 (23.2)	
Friend/classmate/colleague	25 (2.4)	12 (1.7)	
Other (e.g., domestic helper)	16 (1.5)	33 (4.7)	
Frequency of contact with SARS patient within 10 days of hospital admission			0.06
Daily	666 (62.6)	405 (57.9)	
4–6 days per week	82 (7.7)	56 (8.0)	
1–3 days per week	161 (15.1)	103 (14.7)	
Very occasionally	155 (14.6)	135 (19.3)	
No. of precautions adopted during SARS outbreak^b^			0.25
<2	60 (6.6)	47 (8.6)	
3–4	113 (12.4)	81 (14.8)	
5–6	334 (36.7)	187 (34.1)	
7–8	402 (44.2)	234 (42.6)	
No. of febrile or respiratory illness episodes since February 2003			0.02
0	643 (61.7)	471 (68.4)	
1–2	351 (33.7)	193 (28.0)	
>3	48 (4.6)	25 (3.6)	
Presence of chronic medical conditions			0.10
Yes	270 (28.3)	149 (24.6)	
No	683 (71.7)	457 (75.4)	
Self-perceived health status in previous week			0.34
Excellent	124 (11.6)	84 (12.0)	
Very good	317 (29.7)	222 (31.8)	
Good	323 (30.3)	223 (31.9)	
Fair	279 (26.1)	152 (21.7)	
Poor	24 (2.2)	18 (2.6)	

^a^Includes Canada, China, Singapore, and Taiwan. ^b^Includes washing hands before touching mouth, eyes, and nose; washing hands with soap; wearing face mask; using serving utensils during meals; adopting precautionary measures when touching possibly contaminated objects, washing hands after touching possibly contaminated objects; adopting home preventive measures (such as maintaining good ventilation and using bleach to clean surfaces and home appliances) against SARS; and adopting workplace preventive measures (such as maintaining good ventilation, using bleach to clean surfaces and office furniture, and not allowing staff who are sick to come to work) against SARS.

The extent of seroconversion in close contacts of confirmed patients should provide the upper limit of SARS-CoV antibody seroprevalence in the general population, given the relatively intense exposure history of these persons to SARS patients. Our finding of the near absence of transmission resulting in asymptomatic infection in this representative high-risk group of close contacts indicates that the prevailing SARS-CoV strains in Hong Kong almost always led to clinically apparent disease. Whereas some SARS patients, especially healthcare workers, might have been initially admitted to reduce transmission to family members, virtually all SARS patients (perhaps with very few exceptions in children [4]) had severe disease requiring inpatient treatment; thus, we can infer that infection with SARS-CoV inevitably caused severe disease requiring hospitalization.

Although our results suggest that SARS-CoV was a new virus in humans without a close precursor or an antigenically related virus that would have induced at least a small degree of cross-reactivity on serologic testing, a recent study on a select group of 938 healthy Hong Kong adults, whose serum had been previously stored as part of a hepatitis B serosurvey in 2001, indicated that 1.8% of the sample had acquired a SARS-CoV–related virus infection at least 2 years before the 2003 SARS outbreak (*5*). The investigators speculated that the virus that affected these healthy, seropositive persons was antigenically closer to the recently isolated animal SARS-CoV–like virus (*3*) than human SARS-CoV, but interspecies transmission from animals to humans was probably inefficient, as the virus might not have adapted in the new host. This hypothesis would explain why only a few persons became infected and why they were likely to have been asymptomatic. This hypothesis would be compatible with the presumed asymptomatic infection observed in Guangdong animal traders, especially in those who handled masked palm civets, who had a seropositivity rate of 72.7% (exact 95% CI 49.8%–89.3%) in the absence of prior overt clinical disease (*6*).

The limitations of the study include incomplete contact tracing, especially in the earlier parts of the epidemic, and potential recall bias from underreporting of contacts by some patients who were too sick to answer questions. Another possible shortcoming is the lack of a survey of close contacts who did not report a telephone number, although there is no reason to suspect they had a systematically different serologic profile. In fact, these were mostly nonhousehold contacts who would have had less intense exposure to SARS patients. In addition, because peak infectivity, as indicated by viral load, usually occurred during week 2 of illness (*7*), when most of the patients would have been isolated in hospital (the mean onset-to-admission interval decreased from a maximum of 9.3 days in late February to 1.0 day by mid-May) (*8*), transmission to close contacts in the later stages of the epidemic was less likely. Finally, contacts who refused to participate (561) or refused to have serologic testing (708) might have done so because they were concerned about having had SARS (possibly because of having had SARS-like symptoms) and did not want to be identified and stigmatized as having been infected with SARS-CoV. Surveys in other countries with large-scale outbreaks such as Canada, China, Singapore, and Taiwan should be undertaken to confirm our findings.
