# Correction, vol. 10, no. 9

**DOI:** 10.3201/eid1010.C11010

**Published:** 2004-10

**Keywords:** errata, erratum, correction

In “SARS-CoV Antibody Prevalence in All Hong Kong Patient Contacts” by Gabriel M. Leung et al., errors occurred on p. 1654. The seventh sentence read “those who declined testing” but should have been “those who consentedto testing.”

The corrected sentence reads as follows: However, those who consented to testing were more likely to report more frequent contact and closer relationships with SARS patients, more febrile or respiratory illness episodes since February, and a travel history to SARS-affected regions, which may have biased our seroprevalence estimate upwards.

The corrected article appears online at http://wwwnc.cdc.gov/eid/article/10/9/04-0155_article.htm.

 We regret any confusion these errors may have caused.

